# Comparative Diagnostic Performance of Estimated Fetal Weight and Isolated Abdominal Circumference for the Detection of Fetal Growth Restriction

**DOI:** 10.1002/jum.16001

**Published:** 2022-05-03

**Authors:** Megan D. Whitham, David M. Reynolds, Amanda R. Urban, Christopher S. Ennen, Donald J. Dudley

**Affiliations:** ^1^ Department of Obstetrics and Gynecology University of Virginia School of Medicine Charlottesville VA USA

**Keywords:** composite perinatal morbidity, fetal growth restriction, FGR, growth estimates, small‐for‐gestational age

## Abstract

**Objectives:**

To describe the comparative incidence, detection of small‐for‐gestational age (SGA), and composite perinatal morbidity (CPM) associated with diagnostic criteria of fetal growth restriction (FGR) by estimated fetal weight (EFW) <10% with those with isolated abdominal circumference (AC) measurements <10%.

**Methods:**

We performed a retrospective cohort study of 1587 patients receiving prenatal care and delivery at our institution. We included all patients with ultrasounds and delivery outcomes available, and excluded terminations, second trimester losses, and pregnancies without ultrasounds. EFW was calculated from Hadlock and use of the Duryea centiles, and AC from Hadlock's reference curves. We determined SGA at birth and defined CPM as birthweight less than 3% or birthweight less than 10% with neonatal morbidity.

**Results:**

Of 1587 patients, 28 (1.8%) were classified as FGR by EFW <10%. Three of 12 patients with isolated AC <10% developed EFW <10% later in pregnancy (25%). The performance of each diagnostic criteria were comparable for the outcomes of SGA and CPM, with similar sensitivities, but with decreased specificity for SGA outcome, and an increased false positive rate for patients classified as FGR by isolated AC <10, with a tradeoff of decreased false negatives.

**Conclusions:**

Broadening the diagnosis of FGR to include patients with isolated AC <10 did not significantly increase the detection of pregnancies at risk for SGA or CPM. Our conclusions may be limited by a lack of statistical power given a low frequency of SGA and CPM.

**Abbreviations**
CPM: composite perinatal morbidity
EFW: estimated fetal weight
FGR: fetal growth restriction
MFM: maternal‐fetal medicine
SGA: small‐for‐gestational age
U/A Doppler: umbilical artery Doppler

There is controversy regarding the definition of fetal growth restriction (FGR). In October 2020, the Society for Maternal‐Fetal Medicine recommended a change in the diagnosis of FGR to not only include those fetuses with an estimated fetal weight (EFW) <10th percentile (EFW <10%), but also those with an isolated abdominal circumference (AC) measurement <10th percentile (AC <10%).^1^ This definition of FGR is currently employed in the United Kingdom^2^ and was quickly adopted by the American College of Obstetricians and Gynecologists in February of 2021.^3^

Previous studies have concluded that the measurement of AC alone is equivalent to EFW in predicting small‐for‐gestational age (SGA) newborns (birthweight <10th percentile for gestational age)^4^, ^5^ and may have increased sensitivity.^6^ Additional support for expanding the definition was an assertion that isolated AC <10% is uncommon^7^; however, the incidence of this clinical entity is incompletely described.

A limitation of many published studies examining either AC or EFW <10% is that many only report a primary outcome of SGA.^4^, ^8^, ^9^, ^10^ Just as defining fetal growth on centiles by sonographic measurements may incorrectly diagnose pathology in constitutionally small fetuses, this same concern applies to the small, but healthy, neonate. As noted in a recent editorial, while SGA may be correlated with perinatal morbidity, it is a poor discriminator of these outcomes.^11^

The objective of this pragmatic retrospective cohort study is to address whether adopting the diagnosis of FGR to include isolated AC <10% improves accuracy of detecting SGA or composite perinatal morbidity (CPM) and to evaluate population and pregnancy outcome differences for patients diagnosed by these two parameters.

## Materials and Methods

For the purpose of our analyses, we define multiple classifiers. EFW <10% was defined as an EFW measuring less than the 10th percentile during an ultrasound using Hadlock's formula and the Duryea population curve centiles.^12^, ^13^ Patients were categorized as having an isolated AC <10% when an ultrasound measurement of the AC was measured as <10th percentile where the total EFW was measuring ≥10th percentile, with reference to Hadlock population curves. SGA was defined as a neonatal birthweight less than 10th percentile weight for gestational age by Fenton's growth chart.^14^ Criteria for a CPM were met when neonatal birthweight measured less than the third percentile or when the neonatal birthweight was SGA and developed ≥1 concurrent diagnosis including any of the following conditions: respiratory distress syndrome, bronchopulmonary dysplasia, grade III/IV intraventricular hemorrhage, periventricular leukomalacia, blood culture‐positive sepsis, necrotizing enterocolitis, or fetal or neonatal death. Birthweight less than the third percentile was included as a marker of CPM, given significantly increased rates of adverse perinatal morbidity and mortality noted at this degree of growth delay, and as a surrogate of milder morbidity not immediately available from chart review (eg, neonatal ICU admission, resuscitation events, hospital readmissions, and neonatal length of stay).

Our sample population was drawn from pregnant patients at our tertiary care academic medical center with a delivery volume of approximately 2000 deliveries per year. Anatomic surveys and growth ultrasounds for patients planning delivery at the hospital were performed at an outpatient fetal diagnostic unit. We performed a retrospective cohort study of patients with anatomic ultrasounds and/or third trimester growth ultrasounds who delivered during a 2‐year period (January 6, 2018–December 30, 2019). This convenience sample was selected to describe the occurrence of SGA or CPM for all patients whom we would have reasonably had an opportunity to diagnose these conditions over the course of pregnancy and to include patients previously unclassified as FGR who would now meet the new criteria for FGR by isolated AC <10%. All singleton, non‐anomalous pregnancies ongoing beyond the second trimester anatomic surveys who delivered at our tertiary care center were included in our analysis. Patients with early pregnancy loss or termination, or without imaging studies during the incident pregnancy, were excluded. Multiple gestations and patients with major fetal anomalies were likewise excluded from this initial analysis.

At our institution, we employ a risk‐based strategy for obtaining third trimester ultrasound which is common to U.S. academic medical centers. Patients with low‐risk pregnancies are followed in the third trimester by symphysial‐fundal height measurement and a third trimester growth ultrasound is obtained when fundal height is measuring more than 2 cm different than weeks of gestation. For patients at high risk for growth velocity disorders, one‐time or serial third trimester growth ultrasounds are ordered beginning between 28 and 32 weeks gestational age. When serial growth evaluations were performed, a classification of AC <10% or EFW <10% was only obtained based on the results first growth ultrasound performed. All charts were reviewed for additional ultrasound data to assess the frequency at which patients with AC <10% progressed to FGR by EFW <10%.

Hadlock calculations were used to estimate fetal weight^12^ and our institutional practice is to reference the Duryea population‐based growth curve, which is considered less likely to overestimate the incidence of FGR.^13^, ^15^ Hadlock's reference AC curves were used to define AC <10%.^16^ Fetal biometry measurements and umbilical artery Doppler velocimetry studies (U/A Doppler) were performed by 4 certified obstetric sonographers and/or 2 maternal‐fetal medicine (MFM) fellow physicians and interpreted by MFM faculty. Prior to December 2019, these measurements were obtained with General Electric Voluson E8 ultrasound machines, thereafter with Samsung HERA W10 machines. Through the duration of the study, patients were clinically diagnosed with FGR when EFW alone was measuring <10th percentile and these patients were stratified to more frequent monitoring with serial growth ultrasound sonography every 3 to 4 weeks in addition to weekly U/A Doppler and antenatal testing per our institutional protocols. Patients with isolated AC <10% did not receive a specific diagnosis nor alteration of their care plan. U/A Doppler studies were not performed for these patients and the performance of subsequent growth ultrasounds was dependent on individual risk stratification. The timing of delivery was informed by the managing physician and in accordance with the ACOG practice guidelines for management of FGR.^7^ Our study was conducted following the publication of the findings from the Antenatal Late Preterm Steroids trial^17^ and as such, preterm and late preterm antenatal steroids were administered as standard of care when indicated.

Medical, obstetric, ultrasound measurements, delivery details, and neonatal outcomes were manually extracted from records for all eligible pregnancies. All third trimester growth ultrasounds were reviewed for biometric measurements, EFW percentile, and AC percentile. Baseline demographics were described and analyzed for patients with FGR by EFW <10%, isolated AC <10%, and those without concern for FGR complication during the pregnancy by Pearson Chi‐square test, Fisher's exact test, and independent‐samples Kruskal–Wallis analysis where appropriate. Pregnancy outcomes for these three pregnancy designations were similarly analyzed by Pearson Chi‐square test, Fisher's exact test, and independent‐samples Kruskal–Wallis test. When differences were detected between means by independent‐samples test, pairwise comparisons testing for multiple comparisons was performed. Student's *t*‐test was performed for analysis of gestational age at which FGR would have been diagnosed by ultrasound by each diagnostic criteria. Accuracy tests for the detection of SGA and CPM were compared between those patients meeting the historical definition of FGR by EFW <10% or those meeting the new definition of FGR with the addition of patients with isolated AC <10%. The diagnostic sensitivity, specificity, positive predictive value, negative predictive value, and false positive rate for detection of SGA and CPM were compared between these two diagnostic criteria. McNemar's test was performed for comparison of test characteristics of sensitivity and specificity for each outcome of interest. The diagnostic accuracy of a group composed of patients only with isolated AC <10% was not analyzed as no current management schema suggest exclusion of EFW <10% in the diagnosis of FGR. The study was approved by the Institutional Review Board and was determined to be exempt status by the Human Subjects Committee. Statistical analyses were performed using IBM SPSS Statistics for Windows, Version 28.0.

## Results

**Figure 1:**
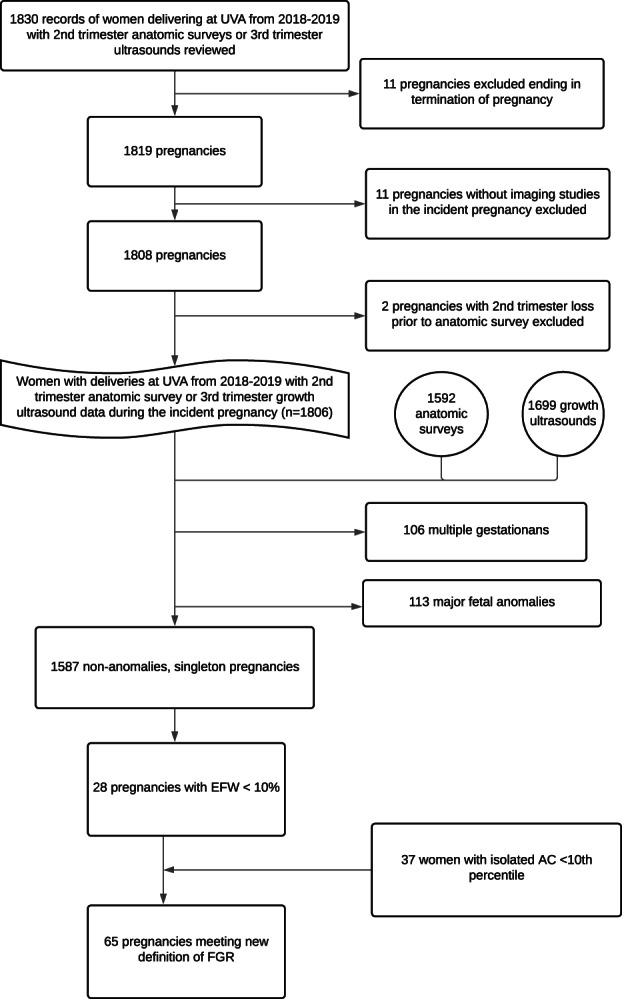
Flowchart of inclusion and exclusion criteria of pregnancies included in evaluation of diagnostic accuracy of EFW (<10%) versus expanded diagnosis with isolated AC (<10%) in predicting pathologic FGR.

One thousand eight hundred thirty records from patients who delivered at our institution and received a charge for an anatomic ultrasound or third trimester growth ultrasound were obtained. Of this sample, 11 pregnancies were excluded due to termination of pregnancy, 11 were excluded without a growth or anatomic ultrasound performed during the pregnancy, and 2 patients were excluded with a second trimester loss prior to the anatomic ultrasound during the pregnancy, leaving 1806 charts for formal review. Our data thus included an initial review of 1592 anatomic surveys and 1699 growth ultrasounds. From the 1806 reviewed charts, 106 were from multiple gestations and 113 pregnancies were complicated by major fetal anomalies, resulting in 1587 non‐anomalies, singleton gestations forming our baseline cohort. From these 1587 pregnancies, 28 (1.8%) patients had a growth ultrasound performed classifying them as FGR by EFW <10%. Another 37 (2.3%) pregnancies would have been additionally categorized as FGR by the new definition with the inclusion of isolated AC <10% (Figure 1) for a total of 65 (4.1%) pregnancies being characterized as FGR by the new criteria of either EFW <10% or isolated AC <10%. Notably of the 37 pregnancies who would have been newly classified as FGR by AC <10%, only 12 patients had additional ultrasounds with 3 (25%) diagnosed as FGR by an EFW <10% later in the pregnancy.

The baseline demographic and clinical characteristics of the included pregnancies are highlighted in Table 1. Consistent with prior research, chronic hypertension and preexisting diabetic status were correlated with the identification of FGR during the pregnancy. There were otherwise no significant differences in baseline characteristics identified in the population of patients who developed FGR by either EFW <10% or AC <10% from patients with normal growth parameters without the evidence of FGR.

**Table 1:** Baseline Demographic and Clinical Characteristics of Study Population

Characteristic	Normal Growth, No FGR (*n* = 1522)	EFW <10% (*n* = 28)	Isolated AC <10% (*n* = 37)	*P*‐Value
Race^a^				
White/Caucasian	1046 (68.7)	16 (57.1)	22 (59.5)	NS (.77)
Black	146 (9.6)	5 (17.8)	4 (10.8)	
Asian	87 (5.7)	4 (14.3)	2 (5.4)	
Other	243 (15.9)	3 (10.7)	9 (24.3)	
Hispanic ethnicity^a^				
Yes	333 (21.9)	4 (14.2)	9 (24.3)	NS (.88)
No	1184(77.8)	24 (85.7)	28 (75.6)	
No data	2 (1.3)			
Smoking status^a^				
Current	99 (6.5)	2 (7.1)	3 (8.1)	NS (.50)
Former	209 (13.7)	7 (25.0)	6 (16.2)	
Never	1214 (79.8)	19 (67.8)	28 (75.7)	
BMI^b^	25.4 [22.3, 30.6]	25.3 [19.8, 29.1]	26.3 [20.7, 32.2]	NS (.65)
Parity^b^	1 [0,2]	1 [0,1]	1 [0,1]	NS (.35)
Maternal age^b^	30 [26, 33]	28 [23, 33]	31 [27,35]	NS (.43)
Chronic hypertension^b^	60 (3.9)	6 (21.4)	8 (21.6)	<.001
Type 1 diabetes^c^	11 (0.7)	1 (3.6)	2 (5.4)	.017
Type 2 diabetes^c^	29 (1.9)	1 (3.6)	3 (8.1)	.039
Gestational diabetes^c^	109 (7.2)	3 (10.7)	2 (5.4)	NS (.68)

Data reported in median [IQR] or as “*n* (%),” unless otherwise stated. NS, not significant.

^a^ Pearson Chi‐squared test.

^b^ Independent‐samples Kruskal–Wallis test.

^c^ Fisher's exact test.

Pregnancy outcomes are presented in Table 2. The gestational age at which FGR would have been detected by isolated AC <10% was not statistically different from the gestational age at which a diagnosis of FGR was made by EFW <10%. Independent‐samples Kruskal–Wallis test was performed to compare the effect of FGR status (no concern, EFW <10% or isolated AC <10%) on birthweight at delivery and gestational age at delivery which identified a statistically significant difference between groups. Further stepwise analysis revealed that there was a statistically significant difference for patients diagnosed by EFW <10 from both patients without FGR detected, and those with isolated AC <10%, with earlier gestational age at delivery and lower mean birthweight recorded for patients diagnosed by EFW <10%. There was alternatively no significant difference in birthweight or gestational age at delivery from patients without suspicion of FGR noted for those patients with AC <10%.

**Table 2:** Pregnancy Outcomes for Pregnancies With Suspected Normal Growth or FGR Suspected by Either EFW <10% or Isolated AC <10%

Outcome	Normal Growth, No FGR (*n* = 1522)	EFW <10% (*n* = 28)	Isolated AC <10% (*n* = 37)	*P*‐Value
GA at which FGR would have been diagnosed^a^	N/A	31.4 [28.5, 34.9]	29.4 [28.2, 32.1]	NS (.07)
Gestational hypertension^b^	110 (7.2)	7 (25.0)^d^	3 (8.1)	.01
Gestational age at delivery (weeks)^c^	39 [38, 40]	37 [35, 38]^d^	39 [36, 39]	<.001
Birthweight at delivery (grams)^c^	3370 [3056, 3694]	2315 [1645, 2828]^d^	2783 [2339, 3070]	<.001
SGA at delivery^b^	63 (4.1)	21 (75.0)^e^	6 (16.2)^d^	<.001
CPM outcome^b^	23 (1.5)	10 (35.7)^d^	2 (5.4)	<.001

Data reported in median [IQR] or as “*n* (%),” unless otherwise stated. NS, not significant.

^a^ Independent‐samples Kruskal–Wallis test with stepwise comparison.

^b^ Fisher's exact test.

^c^ Independent Student's *t*‐test.

^d^ Denotes group with statistical significance from baseline population without concern for FGR.

^e^ Denotes statistical significance from both group without suspected FGR and those with isolated AC.

The development of gestational hypertension was correlated with a diagnosis of FGR by EFW <10%, but residual analysis by Fisher's exact test between groups revealed the development of gestational hypertension was not significantly different between pregnancies with no concern for FGR and those with isolated AC <10%. While the detection of SGA was significantly different for pregnancies with either a diagnosis of EFW <10% or AC <10% compared to pregnancies without suspected FGR, there was a further difference detected in SGA frequency between groups with EFW <10% when compared with pregnancies affected by isolated AC <10%. CPM outcome was correlated with diagnosis of EFW <10%, but not statistically different from patients without suspected FGR for pregnancies affected by isolated AC <10%.

The remainder of our analyses compare all cases that would have been identified as FGR by the new criteria (EFW and/or AC <10%) versus those identified as FGR during the pregnancy by the traditional diagnostic criteria of EFW <10% only.

SGA was detected at birth or at fetal death examination in 90 (5.7%) of pregnancies. The traditional criteria of FGR by EFW <10% identified 21 of these pregnancies at the first growth ultrasound resulting in a sensitivity of 23.3% (15.1, 33.4), specificity of 99.5% (99.0–99.8), positive predictive value of 75.1% (56.7, 87.3), and negative predictive value of 95.6% (95.1, 96.0). By broadening the definition of FGR to include isolated AC <10%, 6 additional cases of SGA would have been correctly identified at the first growth ultrasound for a total of 27 neonates detected with SGA birthweight, resulting in a sensitivity of 30.0% (20.8, 40.6), specificity of 97.5% (96.5, 98.2), positive predictive value of 41.7% (31.3, 52.6), and negative predictive value of 93.6% (95.3, 96.4) (Table 3). McNemar's test was performed to compare the performance of the sensitivities of the two diagnostic criteria, with a significant difference detected, with a Chi‐squared statistic of 6, and *P* = .014.

**Table 3:** EFW <10% Versus New Definition With Inclusion of Isolated AC <10% on Prediction of SGA

Traditional Definition FGR	SGA	No SGA	Total	New Definition	SGA	No SGA	Total
EFW <10th percentile	21	7	28	Isolated AC or EFW <10th	27	38	65
EFW never <10th percentile	69	1490	1559	No diagnosis FGR	63	1459	1522
Total	90	1497	1587		90	1497	1587

CPM was detected at birth or fetal death examination in 35 patients. FGR by EFW <10% identified 10 of these pregnancies at the first growth ultrasound resulting in a sensitivity of 28.6% (14.6, 46.3), specificity of 98.8% (98.2, 99.3), positive predictive value of 35.7% (21.7, 52.7), and negative predictive value of 98.4% (98.0, 98.7). By broadening the definition of FGR to include isolated AC <10%, 2 additional cases of CPM would have been correctly identified at the first growth ultrasound for a total of 12 neonates that would have been detected with resulting CPM at birth. This new definition thus would have resulted in a sensitivity of 34.2% (19.1, 52.2), specificity of 96.6% (95.6, 97.4), positive predictive value of 18.4% (11.8, 27.8), and negative predictive value of 98.5% (94.0, 96.2) for prediction of CPM (Table 4). McNemar's test was performed to compare the performance of the sensitivities of the two diagnostic criteria, and no significant difference was detected for detection of CPM, with a Chi‐squared statistic of 2, and *P* = .157.

**Table 4:** EFW <10% Versus New Definition With Inclusion of Isolated AC <10% on Prediction of CPM

Classic definition FGR	CPM	No CPM	Total	New Definition FGR	CPM	No CPM	Total
EFW <10th percentile	10	18	28	Isolated AC or EFW <10th	12	53	65
EFW never <10th percentile	25	1534	1559	No diagnosis FGR	23	1499	1522
Total	35	1552	1587		35	1552	1587

Overall, we found the new definition of FGR to include isolated AC <10th percentile during the pregnancy would have resulted in the detection of 6 additional cases of SGA and 2 cases of CPM with 38 additional false positives who never developed an outcome of interest.

## Discussion

Our study is a preliminary investigation of outcome differences between populations that would be classified as FGR by traditional criteria of EFW <10% and those who meet the new diagnostic definition of FGR by including isolated AC <10%. Our study population had a low rate of SGA and CPM outcomes, and we are unable to assess the true superiority of one diagnostic criteria over another. In our population, SGA was more common in pregnancies affected by isolated AC <10% than for pregnancies without concern for FGR, but these pregnancies were not at substantially increased risk for the pregnancy complications of gestational hypertension or CPM. Gestational age and birthweight at delivery were also similar between patients classified as FGR by AC <10% and those without suspected FGR.

Numerous prior publications have reported poor detection rates of SGA by third trimester growth ultrasound screening of 20 to 50%.^18^, ^19^, ^20^, ^21^ Similarly, our detection rate for SGA at birth was poor at 23.3% by the traditional definition, increasing only to 30% when including isolated AC <10%, with a significant difference in sensitivity noted by McNemar's test, but with similar confidence intervals. We found that the specificity for detection of SGA would be decreased from 99.5% with traditional criteria to 97.5% with inclusion of patients with isolated AC <10%. The strength of our conclusions for reporting differences in comparative sensitivities is limited due to low frequency of SGA outcome within our population and low detection rates by both diagnostic criteria. At an alpha of 0.05, 6584 patients would need to have been included from a study population with similar characteristics as ours to reach 80% power for such comparisons.

One argument for broadening the definition of FGR is to avoid missing the identification of truly growth‐restricted fetuses at risk for poor neonatal outcomes. Support for this recommendation comes from the contemporary meta‐analysis published by Caradeux et al,^6^ in which the authors demonstrate that at a fixed false positive rate, isolated AC <10th percentile increases the sensitivity of diagnosis of SGA. Unfortunately, the analyzed studies included limited detailed neonatal and longitudinal outcomes data, precluding the analysis of CPM. We provide birth outcomes data and an assessment of the incidence of CPM occurring in an otherwise diverse patient population. We also considered how broadening the diagnostic criteria of FGR may impact the sensitivity, positive predictive value, and false‐positive rates of CPM. Notably, less than half (38.8%) of pregnancies ultimately diagnosed as SGA were at risk for a CPM and overall detection of CPM was comparatively poor for both traditional diagnostic criteria and by expanding the diagnostic criteria to include pregnancies with isolated AC <10%. Sensitivities and specificities of these tests were similar, and we did not detect a statistically significant difference in the function of these diagnostic criteria for detection of CPM by McNemar test. In line with the limitations of our conclusions at drawing comparisons between the two diagnostic criteria seen with the detection of SGA, we lack statistical power to detect significant differences in the detection of CPM due to the rarity of this clinical entity. Additional prospective studies inclusive of a large cohort would be critical in being able to make stronger conclusions about the diagnostic performance of these tests.

Prior research by Monier et al shows misdiagnosis of FGR results in increased risks of provider‐initiated deliveries prior to 39 weeks.^22^ Our data indicate that expanding the definition of FGR to include isolated AC <10% may result in an increased false‐positive rate. In larger populations, should the finding hold, there may be increased risks for iatrogenic delivery prior to 39 weeks on a larger scale, which carries fiscal and psychosocial costs.^23^ Alternatively, expanding the definition of FGR to include patients with AC <10% increased the detection of both SGA and CPM cases, resulting in a reduction of false negatives where the fetus and neonate may be at risk for adverse perinatal events or stillbirth. We acknowledge that our conclusions are hypothesis generating and that a comparison of the risks and benefits of using either diagnostic criteria is highly dependent on value systems and must include consideration of the cost of care, stress of misdiagnosis, and potential for iatrogenic harm, weighed against the risk of undiagnosed pathology or stillbirth. From the standpoint of these considerations, we are unable to conclude the superiority of either diagnostic approach for these ends based on our findings within this study population.

A strength of our study is the inclusion of all pregnancies with second and third trimester growth ultrasound data delivered at our institution. This allowed for an examination of how broadening the diagnostic criteria of FGR would alter detection in a general American population. Our study also adds to the literature by reporting longitudinal ultrasound data, fetal death, and neonatal and pregnancy outcomes data, allowing us to provide diagnostic performance analyses of both diagnostic criteria for the development of SGA as well as CPM.

Our study has limitations. The data were collected from a single‐center institution and a convenience sample was chosen. This sampling method was purposely selected to acquire direct ultrasound measurement data that were not readily obtainable solely from querying the electronic health record. Our cohort included a low prevalence of both SGA and CPM outcomes and likely represents a relatively healthy cohort, which may limit the generalizability of our outcomes to other centers. Our retrospective design also precludes causal analysis of factors responsible for SGA or CPM, and conclusions about the analysis of pregnancy outcomes must also be limited in scope due to pragmatic design of the study (by convention, those patients meeting FGR criteria by EFW <10 would have been assigned to more ultrasounds, versus patients without this diagnosis, including those with isolated AC <10 would have been assigned to fewer ultrasounds).

Isolated AC <10% and EFW <10% perform similarly in the detection of SGA and CPM, and detection rates remain suboptimal. Large prospective cohort evaluation of these diagnostic criteria should be planned for thoughtful consideration of the benefits and risks associated with either approach.
